# Serum C-reactive protein to albumin ratio as a potential risk indicator of pneumonia caused by *Chlamydia psittaci*: a multicenter retrospective study

**DOI:** 10.3389/fcimb.2024.1371625

**Published:** 2024-06-26

**Authors:** Tianyun Shi, Yunxia Yu, Yao Shen, Meili Shen, Yong Du, Meng Zhang, Zhoufang Mei, Yi Ding, Jingjing Feng, Moran Zhu, Fangxia Ge, Qi Zhao, Ruilan Wang, Zhijun Jie

**Affiliations:** ^1^ Department of Pulmonary and Critical Care Medicine, Shanghai Fifth People’s Hospital, Fudan University, Shanghai, China; ^2^ Center of Community-Based Health Research, Fudan University, Shanghai, China; ^3^ Department of Pulmonary and Critical Care Medicine, Shanghai Pudong Hospital, Shanghai, China; ^4^ Medical Department, Nanjing Dinfectome Technology Inc., Nanjing, China; ^5^ Department of Critical Care Medicine, Shanghai General Hospital, Shanghai Jiaotong University, School of Medicine, Shanghai, China

**Keywords:** C-reactive protein, albumin, C-reactive protein to albumin ratio, *Chlamydia psittaci*, pneumonia

## Abstract

**Introduction:**

The aim of the study was to describe psittacosis pneumonia and to assess the predictive value of the C-reactive protein/albumin ratio in psittacosis pneumonia for severity.

**Methods:**

Data on psittacosis pneumonia cases diagnosed using metagenomic sequencing were collected from three hospitals in Shanghai, China from Oct. 2019 to Oct. 2022. Serum levels of C-reactive protein and albumin were measured and the C-reactive protein to albumin ratio (CAR) was calculated. Spearman’s correlation analysis, ordered logistic regression analysis, and receiver operating characteristic curve analysis were conducted to examine the correlation and predictive ability of the three indicators on the severity of the disease.

**Results:**

A total of 27 patients with psittacosis pneumonia were enrolled, with an average age of 62 years and 70.4% being male. 44.4% of patients had a clear history of contact with poultry or birds. The predominant symptom was fever (100%). Patients treated in the respiratory intensive care unit (RICU) had a higher likelihood of experiencing wheezing (88.9% versus 33.3%, P=0.013) and chest tightness (88.9% vs. 33.3%, P=0.013) than those in the general ward (Non-RICU). The proportion of patients with pleural effusion was significantly higher in the RICU compared to the Non-RICU (88.9% vs. 38.9%, P=0.019). The RICU group had a significantly higher CAR than the Non-RICU group (9.41 vs. 4.05, P=0.017). This result was accompanied by higher intubation and ventilator support (33.3% vs. 0.0%, P=0.029), higher PCT and CRP levels and lower albumin and PaCO2 levels in the RICU than in the Non-RICU. Logistic regression analysis indicated that CAR (OR 1.49; 95% CI 1.07–2.06, P=0.017) was risk factor for prolonged hospitalization (> 14 days).

**Discussion:**

Elevated serum CAR levels were found to be associated with a greater risk of severe psittacosis pneumonia. Consequently, it may serve as an uncomplicated and useful diagnostic tool for clinicians to promptly and precisely ascertain the severity of psittacosis pneumonia, ultimately aiding them in devising the most optimal therapeutic plan.

## 1 Introduction

Psittacosis is a zoonotic infectious disease caused by the transmission of *Chlamydia psittaci* (Yang et al., 2022); it often presents as a pulmonary infection but rarely as a disseminated disease. Psittacosis pneumonia can be caused by inhaling dust from the excreta of birds such as canaries, parrots, pigeons and turkeys. Recently it has been reported that peacocks and chickens can also carry the pathogen and cause infection. Therefore, people who have a long-term preference for eating poultry, workers who raise poultry on farms, vendors who sell poultry at vegetable markets, or people who keep pet birds, are all high-risk groups for infection with *Chlamydia psittaci*. Patients infected with psittacosis will have symptoms such as weakness, diarrhea, impaired liver function and weight loss. Furthermore, they may experience headaches, chills, fever, malaise, myalgia, dry cough and breathlessness. In some severe cases, patients may even die (Branley et al., 2014).

Diagnostic methods currently employed for *Chlamydia psittaci* include culture of the pathogen, serological detection and molecular biology techniques such as PCR. Psittacosis is effortlessly underdiagnosed and misdiagnosed, due to its non-specific symptoms and the restrictions of present tests. Metagenomic next-generation sequencing (mNGS) is a culture-independent technology that has been widely applied in microbiology research and more recently has been increasingly used in clinical diagnostics (Tang et al., 2022). It can help the diagnosis of respiratory diseases including psittacosis pneumonia.

Serum C-reactive protein (CRP) is a hepatic acute-phase reactant that functions as an early marker for inflammation and infection. CRP levels rise rapidly, typically peaking within 48 hours of disease onset. In addition, albumin, another hepatocyte product, correlates significantly with inflammation and nutritional status as it decreases in plasma (Rathore et al., 2022). The C-reactive protein to albumin ratio (CAR), a novel biomarker, has been proposed as a more reliable risk indicator for inflammatory conditions compared to CRP or albumin alone (Kim et al., 2015). Study shows that the high value of CAR was associated with poor outcome in various diseases, including sepsis (Liu et al., 2023), lung cancer (Miyazaki et al., 2022), and some chronic inflammatory disease of the airways (Li et al., 2021). A recent study found that elevated levels of serum CAR and CRP in middle-aged and older Finnish men were each associated with an increased risk of pneumonia (Kunutsor and Laukkanen, 2022). However, the association between CAR and psittacosis pneumonia has not been previously investigated.

In this retrospective analysis, 27 patients diagnosed with psittacosis pneumonia and admitted to either RICU or Non-RICU were enrolled. The study aims to investigate the prognostic significance of CAR in evaluating the severity of psittacosis pneumonia thoroughly.

## 2 Materials and methods

### 2.1 Ethics statement

This work was approved by the Human Research Committee at the Shanghai Fifth People’s Hospital, Fudan University (Approval No.2022–107), and written informed consent was obtained from the patients or their families.

### 2.2 Study design

Clinical data from 27 patients with psittacosis pneumonia, who were admitted to Shanghai Fifth People’s Hospital, Fudan University, Shanghai Pudong Hospital and Shanghai General Hospital between October 2019 and October 2022, were retrospectively analyzed in this multicenter study. The diagnosis for all cases was confirmed through mNGS in combination with clinical manifestations, radiological chest examination, and other laboratory diagnostic methods. mNGS was indicated when a patient met the conditions recommended by expert consensus (Chinese_Thoracic_Society, 2023), and provided informed consent for testing. Blood and sputum samples or bronchoalveolar lavage fluid (BALF) were collected for mNGS. The clinical data of each patient were obtained from the electronic medical record system. The laboratory indicators and CT images were collected within 24–48h after the patient’s admission to the hospital and RICU. The albumin concentration was measured on the Roche Cobas c702 automatic biochemical analyzer using bromocresol green method. The CRP was measured on the Siemens BNIIautomatic protein analyzer. The patients were classified into two groups based on the disease severity, which was determined by the place of treatment (RICU or Non-RICU). The RICU groups fulfilled the diagnostic criteria for severe pneumonia specified by the American Thoracic Society and Infectious Diseases Society of America (Metlay et al., 2019).

### 2.3 Metagenomic next-generation sequencing and pathogen diagnosis

Blood samples were utilized to prepare plasma, and circulating cell-free DNA (cfDNA) was isolated from plasma with the QIAamp Circulating Nucleic Acid Kit (Qiagen, German) in accordance with the manufacturer’s protocols. Sputum was liquefied by 0.1% DTT (dithiothreitol) for 20 minutes at 56°C prior to extraction. Sputum and BALF DNA were then extracted using the TIANamp Magnetic DNA Kit (TIANGEN, China) according to the manufacturer’s protocol. The quantity and quality of DNA were assessed using the Qubit (Thermo Fisher Scientific, USA) and NanoDrop (Thermo Fisher Scientific, USA), respectively. DNA libraries were prepared using the Hieff NGS OnePot II DNA Library Prep Kit for MGI (Yeasen Biotechnology, China) according to the manufacturer’s instructions. Agilent 2100 was used for quality control and DNA libraries were 50bp single-end sequenced on DIFSEQ-200 (Dinfectome, China). No template negative controls (NTCs) were included in the extraction, library preparation, and sequencing process.

Raw sequencing data was splitted by bcl2fastq2 (version 2.20), and high-quality sequencing data were generated by removing low quality reads, adapter contamination, duplicated and shot (<36 bp) reads. Human host sequences were identified by mapping to human reference genome (hs37d5) using bowtie2 software (version 2.2.6). Reads that could not be mapped to the human genome were retained and aligned with microorganism genome database for pathogens identification. Our microorganism genome database contained bacteria, fungi, virus and parasite genomic sequences (download from https://www.ncbi.nlm.nih.gov/). The criteria for positive results of mNGS were as followed: (1) For Mycobacterium, Nocardia and Legionella pneumophila, the result was considered positive if a species detected by mNGS had a species-specific read number ≥1. (2) For bacteria (excluding Mycobacterium, Nocardia and Legionella pneumophila), fungi, virus and parasites, the result was considered positive if a species detected by mNGS had at least 3 non-overlapping reads. (3) Pathogens detected in the NTC were excluded unless the reads number was ≥10-fold than that in the NTC. In this retrospective analysis, we further differentiated between pathogenic and background microorganisms for mNGS-positive results referred to literature reports of respiratory commensal flora and mNGS consensus interpretation principles. A microorganism with species-specific readings ≥1 were also considered positive if it was consistent with clinical diagnosis by reviewing the original detection information.

Clinical microbiological tests included smear, acid-fast staining (AFS) and culture, 1,3-β-D glucan (BDG) test and galactomannan (GM) test. Respiratory viruses and atypical pathogen were detected by PCR or Elisa. In general, pathogen diagnosis was prioritized in cases where a microorganism was detected by both mNGS and clinical microbiological tests. Candidate infectious agents were identified by two experienced clinicians. This identification was based on epidemiological considerations, the immune status of the host, clinical presentation, laboratory findings, chest radiological findings, and therapeutic efficacy.

### 2.4 Statistical analysis

All statistical analyses were conducted using IBM SPSS 25.0 and GraphPad Prism 9.4.1. In instances where continuous variables conformed to normal distribution, the means ± standard deviation (SD) were displayed, whereas the median [interquartile range, (IQR)] was presented for non-conforming variables. For categorical data, frequencies and percentages were utilized for display purposes. Data meeting the assumptions of normality and homogeneity of variance were compared via two-tailed Student’s t-tests, while the Welch correction was applied to data that did not conform to the normality assumption as evaluated by the homogeneity of variance test. Data not meeting the normality assumption were subjected to Mann-Whitney U tests. Categorical variables were analyzed using either Pearson chi-square or Fisher exact tests. The study conducted Spearman’s correlation analysis to examine the correlations between CRP, albumin, and CAR in the two groups. The regression analysis of CRP, CAR, and days in the hospital was analyzed, along with the corresponding P value calculated by Pearson’s correlation analysis. Furthermore, logistic regression was used to identify independent prognostic factors for patients with hospitalization longer than 14 days. The predictive accuracy of prognostic factors was evaluated by using receiver operating characteristic (ROC) curves and area under the curve (AUC). A two-tailed P-value <0.05 was considered statistically significant.

## 3 Results

### 3.1 Demographic, clinical information and imaging features

**Figure 1:**
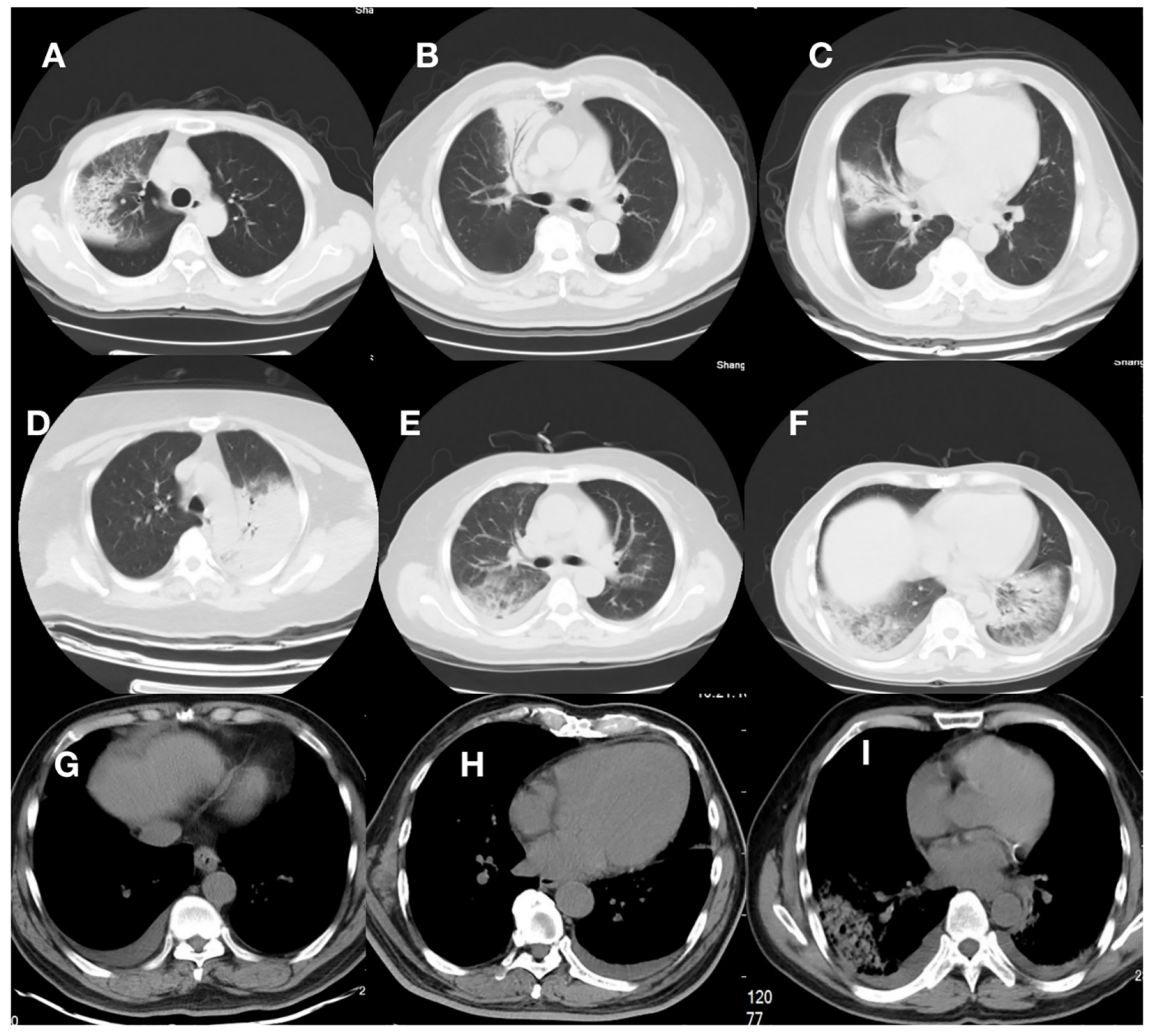
Computed tomographic images from 9 patients. **(A–D)**, and **(G)** were patients in the Non-RICU group, while **(E, F)** and **(H, I)** were patients in the RICU group. **(A–D)**: CT scan showed right-side or left -side pneumonia; **(E, F)**: CT scan showed bilateral pneumonia. **(G–I)**: CT scan showed pleural effusion. Consolidation and patchy shadowing with the air bronchogram sign and ground-glass opacities can be found in the images.

During the study period, 27 patients were eligible for this analysis (**Table 1**), of whom 9 were treated in the RICU and 18 in the general ward (Non-RICU). Psittacosis pneumonia was eventually confirmed by mNGS etiology (**Table S1**). A total of 23, 2, and 4 cases of *Chlamydia psittaci* were identified in BALF, sputum, and blood, respectively. In two patients, *Chlamydia psittaci* was detected in both BALF and blood samples, suggesting a serious infection. Both patients were admitted to the RICU. The mean age of the cohort was 62.0 ± 11.32 years, and 19 (70.4%) patients were male. Among the patients, 8 (29.6%) had a smoking history, and 12 (44.4%) had clear contact with poultry. The average duration between symptom onset and hospitalization was 6.26 ± 4.20 days. While there was no significant difference (P=0.153) in hospital stay days between the two groups, the RICU group had a mean time of 17 ± 10.71 days, which was longer than the 9 ± 4.31 days in the general ward (Non-RICU) group. The occurrence of intubation and ventilator support was significantly higher in the RICU group than in the general ward (Non-RICU) group (33.3% vs. 0.0%, P=0.029). Comorbidities comprised hypertension (33.3%), diabetes(11.1%), coronary heart disease (7.4%) and other disease (40.7%). The most prevalent symptoms and signs were fever (100%), cough (85.2%), high fever (81.5%), fatigue (77.8%), sputum (66.7%), pulmonary rales (55.6%), wheezing (51.9%), chest tightness (51.9%), haziness of spirit-mind (29.6%), and myalgias (14.8%). Patients treated in the RICU were significantly more likely to experience wheezing (88.9% vs. 33.3%, P=0.013) and chest tightness (88.9% vs. 33.3%, P=0.013) compared to those treated on the general ward (Non-RICU). A significant difference in lung CT inflammation site was observed between the two groups (P=0.049), with bilateral pneumonia being more prevalent in RICU patients while most patients on the general ward (Non-RICU) had right-side pneumonia. The percentage of patients with pleural effusion was also markedly higher in the RICU than the general ward (88.9% vs. 38.9%, P= 0.019; **Figure 1**).

**Table 1:** Baseline characteristics of the study population.

Characteristics	Total(n=27)	RICU Group (n = 9)	Non-RICU Group (n= 18)	*P* Value*
Sex(male, %)	19(70.4%)	5(55.6%)	14(77.8%)	0.375
Age (years) ± SD	62.04 ± 11.32	62 ± 10.69	65 ± 11.91	0.714
Smoking history(%)	8(29.6%)	2(22.2%)	6(33.3%)	0.676
BMI (kg/m2) ± SD	25.25 ± 3.48	23.96 ± 3.50	25.48 ± 3.53	0.436
Poultry contact history				0.443
No(%)	9(33.3%)	2(22.2%)	7(38.9%)	
Yes(%)	12(44.4%)	4(44.4%)	8(44.4%)	
Unknown(%)	6(22.2%)	3(33.3%)	3(16.7%)	
Time from illness onset to first hospital admission (days) ± SD	6.26 ± 4.20	7 ± 4.80	5 ± 3.71	0.130
Hospital stay (days) ± SD	13.15 ± 7.43	17 ± 10.71	9 ± 4.31	0.153
Intubate and ventilator support (%)	3(11.1%)	3(33.3%)	0(0.0%)	**0.029**
Comorbidities				
Hypertension(%)	9(33.3%)	2(22.2%)	7(38.9%)	0.667
Diabetes(%)	3(11.1%)	0(0.0%)	3(16.7%)	0.529
Coronary heart disease(%)	2(7.4%)	0(0.0%)	2(11.1%)	0.538
Others(%)	11(40.7%)	3(33.3%)	8(44.4%)	0.692
Symptoms and signs				
Cough(%)	23(85.2%)	7(77.8%)	16(88.9%)	0.582
Sputum(%)	18(66.7%)	6(66.7%)	12(66.7%)	1.000
Blood-stained sputum(%)	1(3.7%)	1(11.1%)	0(0.0%)	0.333
Fever(%)	27(100%)	9(100%)	18(100%)	/
Wheezing(%)	14(51.9%)	8(88.9%)	6(33.3%)	**0.013**
Chest tightness(%)	14(51.9%)	8(88.9%)	6(33.3%)	**0.013**
Haziness of spirit-mind(%)	8(29.6%)	4(44.4%)	4(22.2%)	0.375
Fatigue(%)	21(77.8%)	8(88.9%)	13(72.2%)	0.628
Myalgias (%)	4(14.8%)	1(11.1%)	3(16.7%)	1.000
Body temperature				
T<37°C(%)	1(3.7%)	0(0%)	1(5.6%)	1.000
38°C>T≥37°C(%)	0(0%)	0(0%)	0(0%)	/
39°C>T≥38°C(%)	4(14.8%)	2(22.2%)	2(11.1%)	0.582
T≥39°C(%)	22(81.5%)	7(77.8%)	15(83.3%)	1.000
Pulmonary rales(%)	15(55.6%)	6(66.7%)	9(50%)	0.683
Lung CT inflammation site				**0.049**
Left side(%)	9(33.3%)	3(33.3%)	6(33.3%)	
Right side(%)	13(48.1%)	2(22.2%)	11(61.1%)	
Bilateral(%)	5(18.5%)	4(44.4%)	1(5.6%)	
Pleural effusion(%)	15(55.6%)	8(88.9%)	7(38.9%)	**0.019**
Prognosis				0.128
Recovery(%)	25(92.6%)	8(80%)	17(100%)	
Death(%)	2(7.4%)	2(20%)	0(0%)	

*Those with P value < 0.05 were highlighted using the font bold. Data are presented as the number of patients (%), mean ± SD. BMI, body mass index; CT, computerized tomography; T, temperature.

### 3.2 Laboratory results

**Figure 2:**
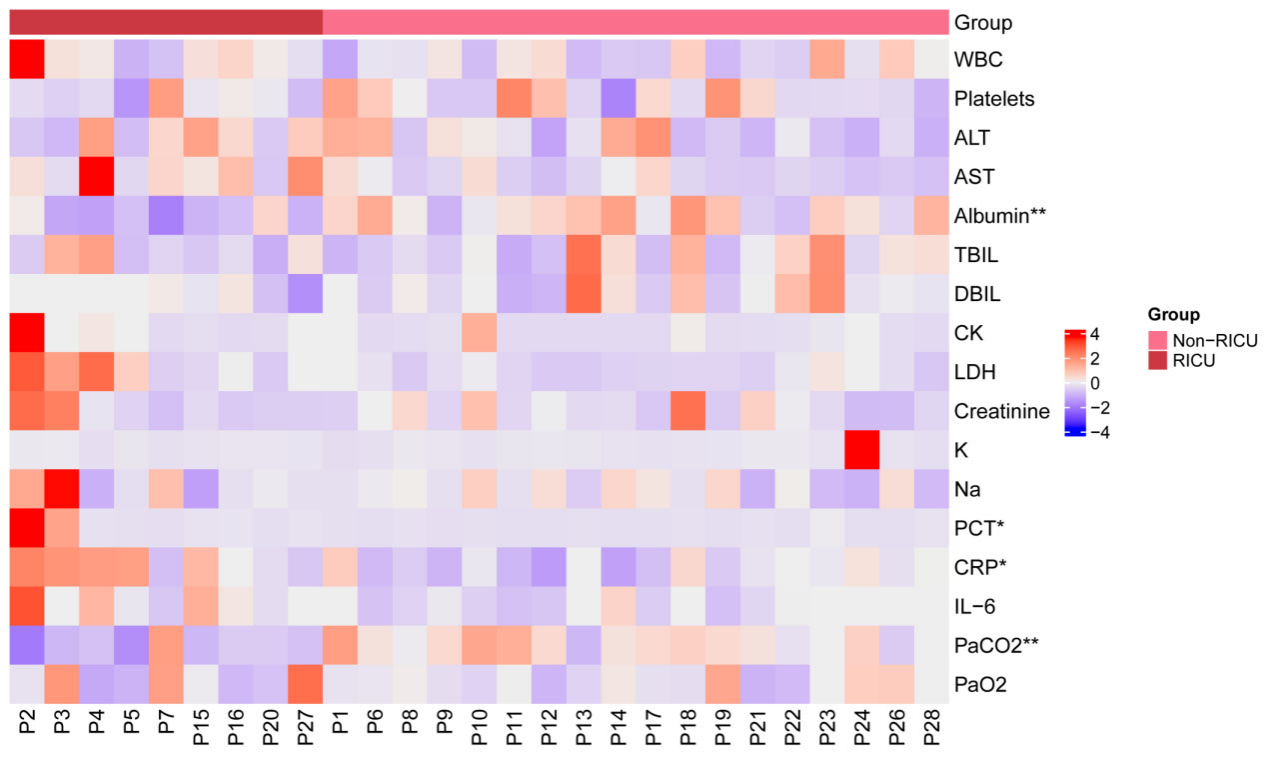
Heatmap showing clinical blood test indicators for 27 patients. The x-axis represents the patient, with red representing the RICU group and pink representing the Non-RICU group. The y-axis shows clinical blood test indicators. These values were standardized (Z-score) for 27 clinical indicators, with redder patches representing higher expression and bluer patches representing lower expression. Statistical significance was determined between the RICU and Non-RICU groups, denoted by *p < 0.05 and **p < 0.01. RICU, respiratory intensive care unit; WBC, white blood cell; ALT, alanine aminotransferase; AST, aspartate aminotransferase; TBIL, total bilirubin; DBIL, direct bilirubin; CK, creatine kinase; LDH, lactate dehydrogenase; K, potassium; Na, sodium; PCT, procalcitonin; CRP, C-reactive protein; IL-6, interleukin-6; PaCO2, arterial partial pressure of carbon dioxide; PaO2, arterial oxygen pressure.

As indicated in **Table 2**, the mean white blood cell (WBC) count of the 27 patients was 6.51×10^9^/L. The mean concentration of alanine aminotransferase (ALT) was 96.74U/L, aspartate aminotransferase (AST) was 146.74U/L, total bilirubin was 13.96 μmol/L, direct bilirubin was 7.59 μmol/L, creatine kinase (CK) was 149.2U/L, lactate dehydrogenase (LDH) was 583.71U/L, creatinine was 92μmol/L, respectively. Albumin and arterial partial pressure of carbon dioxide (PaCO2) levels showed a significant decrease in RICU compared to Non-RICU patients, while procalcitonin (PCT) and CRP levels increased significantly. **Figure 2** highlighted the differences between the two groups more straightforwardly. Median analysis (IQR) revealed that RICU patients exhibited a higher CAR compared to those in the general ward (9.41 [3.42, 11.45] vs. 4.05 [1.96, 4.66], P=0.017). Therefore, an elevated CAR value demonstrated a significant correlation to the severity of psittacosis pneumonia.

**Table 2:** Laboratory tests between RICU group and Non-RICU group patients.

Laboratory Test (Reference Range)	Total(n=27)	RICU Group (n = 9)	Non-RICU Group (n= 18)	*P* Value*
White blood cell(4.00–10.00×10^9^/L) ± SD	6.51 ± 3.03	6.99 ± 4.26	5.76 ± 2.13	0.130
Blood platelet(100.00–300.00×10^9^/L) ± SD	153.04 ± 47.65	137.00 ± 41.93	136.50 ± 50.46	0.401
ALT(<41.00U/L) ± SD	96.74 ± 61.90	126 ± 63.96	78 ± 61.60	0.442
AST(<40.00 U/L) ± SD	146.74 ± 160.48	199 ± 225.16	63 ± 69.27	0.050
Albumin(35.00–50.00g/L) ± SD	33.97 ± 4.07	30 ± 2.84	35.5 ± 3.55	**0.001**
Total bilirubin(<21.00μmol/L) ± SD	13.96 ± 6.79	10.9 ± 6.55	12.6 ± 7.08	0.755
Direct bilirubin(<5.00μmol/L) ± SD	7.59 ± 4.56	6.7 ± 3.57	7.3 ± 4.72	0.253
CK(<190.00U/L)	149.2(71.75,399.5)	198.5(80.5,4311)	149.2(63.25,307.75)	0.461
LDH(135.00–225.00U/L) ± SD	583.71 ± 591.59	780 ± 858.60	318.5 ± 143.58	0.056
Creatinine(45.00–104.00μmol/L) ± SD	92 ± 56	64.5 ± 74.32	72.4 ± 46.3	0.600
Serum potassium (3.50–5.50mmol/L) ± SD	4.96 ± 7.50	3.7 ± 0.46	3.52 ± 9.19	0.514
Serum sodium (135.00–145.00mmol/L) ± SD	136.66 ± 6.31	135 ± 9.79	135.5 ± 3.55	0.399
PCT(0.00–0.50ng/ml)	0.56(0.14,1.26)	1.26(0.42,20.08)	0.31(0.1,0.86)	**0.016**
CRP(<5.00mg/L) ± SD	160.16 ± 96.86	273 ± 116.86	137.88 ± 59.68	**0.023**
IL-6(≤5.40pg/ml) ± SD	118.96 ± 144.04	145.25 ± 188.11	42.85 ± 60.49	0.073
PaCO_2_(35.00–45.00mmHg) ± SD	33.4 ± 5.62	29 ± 5.85	36 ± 3.99	**0.003**
PaO_2_(80.00–100.00mmHg) ± SD	82.64 ± 21.96	78 ± 31.63	77.5 ± 14.79	0.536
mNGS sequence	51(18,528)	211(21.5,5337.5)	47.5(16.75,332.25)	0.231
CAR	4.21(2.56,5.39)	9.41(3.42,11.45)	4.05(1.96,4.66)	**0.017**

*Those with P value < 0.05 were highlighted using the font bold. Data are presented as the number of patients (%), mean ± SD. ALT, alanine aminotransferase; AST, aspartate aminotransferase; CK, creatine kinase; LDH, lactate dehydrogenase; PCT, procalcitonin; CRP, C-reactive protein; IL-6, interleukin-6; PaCO2, arterial partial pressure of carbon dioxide; PaO2, arterial oxygen pressure; mNGS, metagenomic next-generation sequencing; CAR, CRP to albumin ratio.

### 3.3 Correlation analysis between CRP, albumin, CAR and hospitalization days

**Figure 3:**
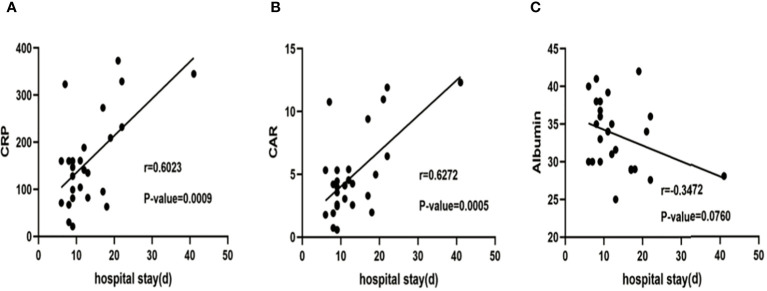
Correlation between CRP **(A)**, CAR **(B)** ,albumin **(C)** and hospitalization days. The line depicts the outcomes of a linear regression analysis, and the P-value was established through Pearson correlation analysis. CRP, C-reactive protein; CAR, C-reactive protein to albumin ratio.

A significant correlation was observed between CRP, albumin, and CAR between the two groups (**Table 3**). **Figure 3** presents the results of a linear regression analysis on the correlation between CRP, CAR, albumin and hospitalization days. Logistic regression analysis indicated that CRP (OR 1.01; 95% CI 1.00–1.03, P=0.016) and CAR (OR 1.49; 95% CI 1.07–2.06, P=0.017) were risk factors for prolonged hospitalization (> 14 days) (**Table 4**). A negative correlation was observed between albumin and RICU (r = -0.606, P = 0.001, **Table 3**), but logistic regression revealed that albumin was not a statistically significant correlation with hospitalization days (r = -0.3472, P = 0.076, **Figure 3**).

**Table 3:** Correlation analysis between CRP, albumin and CAR between the two groups.

	r	*P* Value*
CRP	0.424	**0.028**
Albumin	-0.606	**0.001**
CAR	0.469	**0.014**

*Those with P value < 0.05 were highlighted using the font bold. CRP, C-reactive protein; CAR, C-reactive protein to albumin ratio.

**Table 4:** Logistic regression analysis of CRP, CAR and prolonged hospitalization (> 14days).

	B	Wald	*P* Value*	OR	95%*CI*
CRP	0.014	5.825	**0.016**	1.014	1.003–1.026
CAR	0.396	5.688	**0.017**	1.487	1.073–2.059

*Those with P value < 0.05 were highlighted using the font bold. CRP, C-reactive protein; CAR, C-reactive protein to albumin ratio.

**Figure 4:**
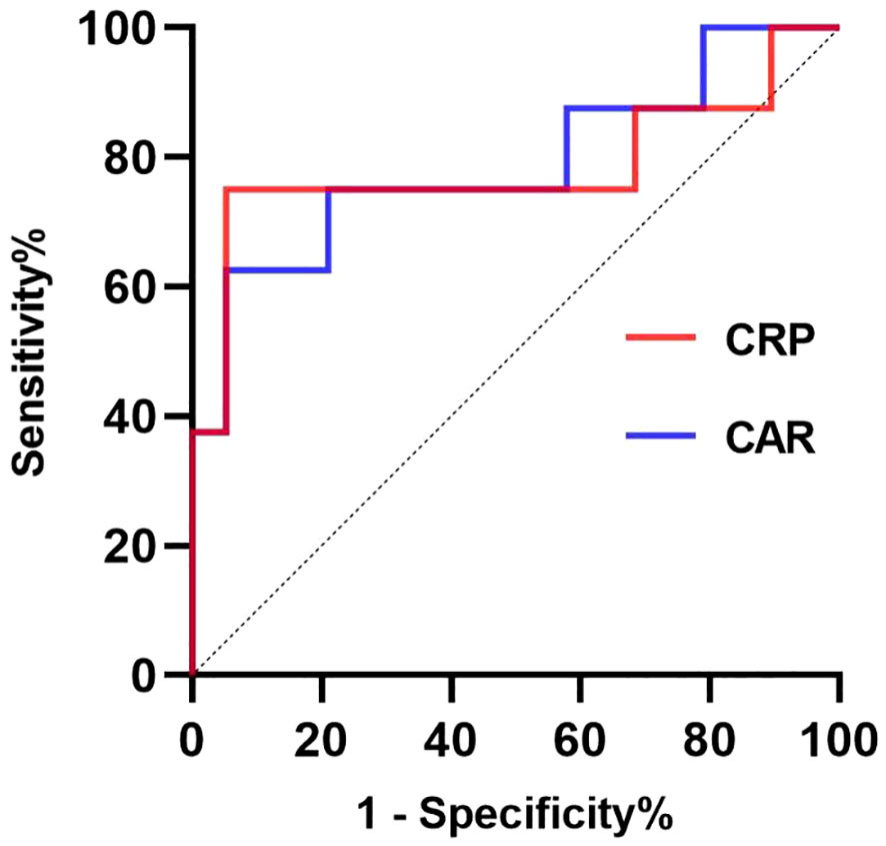
The receiver operating characteristic (ROC) curves for prognostic factors predicting long hospitalization (>14 days). The ROC curve for CRP is presented in the red line; the ROC curve for CAR is presented in the blue line. CRP, C-reactive protein; CAR, C-reactive protein to albumin ratio.

Using a cut-off value of 198.74 mg/L, CRP had a sensitivity of 75% and a specificity of 94.7% for predicting risk factors associated with long hospitalization, yielding an AUC of 78.29% (95% CI 54.14 -100.00%, P=0.0224; **Figure 4**, **Table 5**). At a cut-off of 5.91, CAR had a sensitivity of 62.5% and a specificity of 94.7% for the prognostic factors of long hospitalization, yielding an AUC of 78.95% (95% CI 57.56–100.00%, P=0.0195; **Figure 4**, **Table 5**).

**Table 5:** Cut-offs, sensitivities, and specificities of prognostic factors for prolonged hospitalization (> 14days).

	Cut-off value	Sensitivity (%)	Specificity (%)	AUC	95%CI	*P* Value*
CRP	198.74	0.75	0.947	0.7829	0.5414 -1.000	**0.0224**
CAR	5.9148	0.625	0.947	0.7895	0.5756 - 1.000	**0.0195**

*Those with P value < 0.05 were highlighted using the font bold. CRP, C-reactive protein; CAR, CRP to albumin ratio; AUC, the areas under the curve.

### 3.4 Profile of pathogens

**Figure 5:**
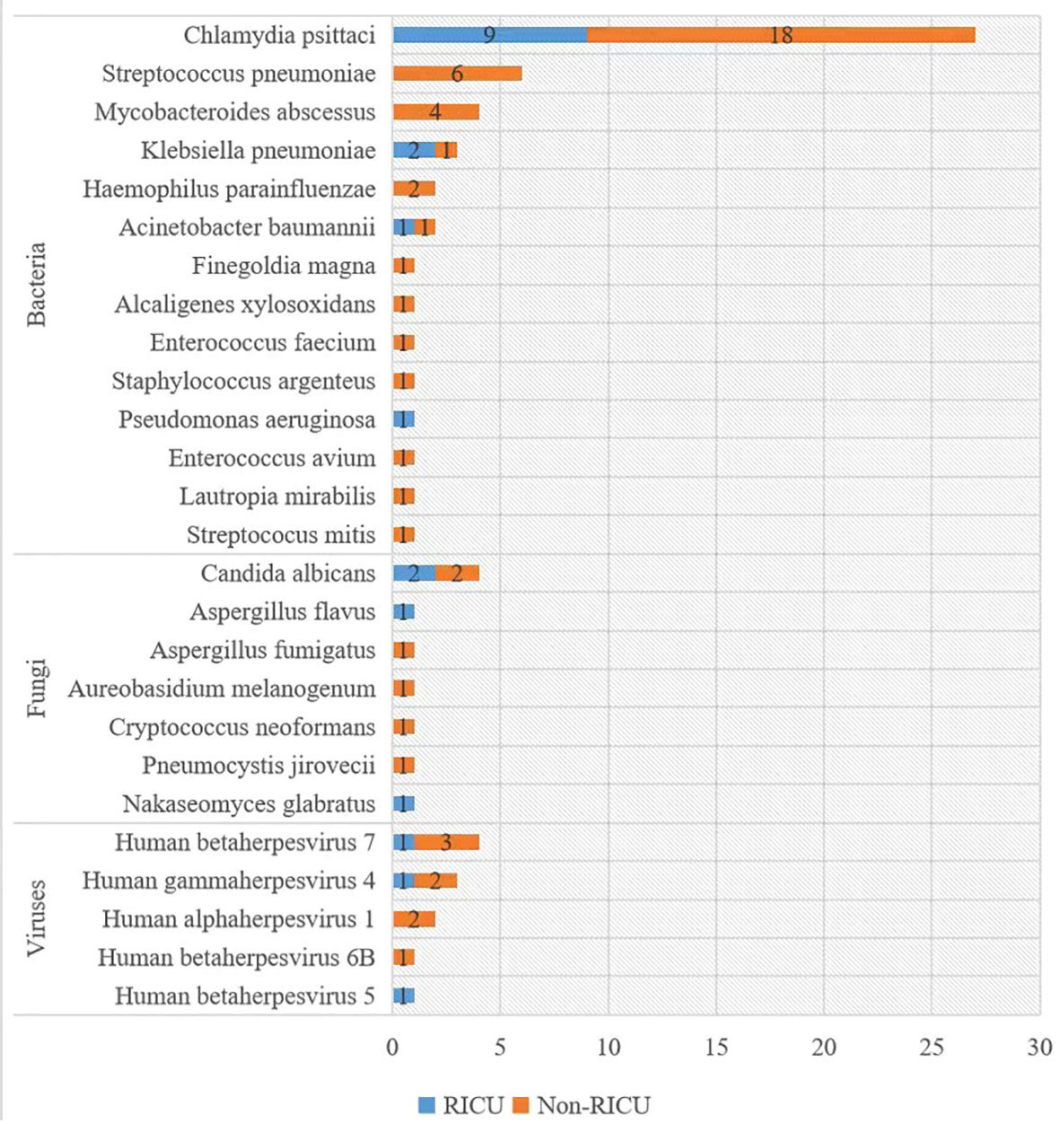
Distribution of pathogens detected by mNGS. The y-axis shows pathogens detected by mNGS, and the x-axis shows the case number. mNGS: metagenomic next-generation sequencing.

**Figure 5** displays the pathogen profile of 27 patients with psittacosis pneumonia using mNGS. A total of 14 bacteria, 7 fungi, and 5 viruses were identified as candidate pathogens using mNGS. In addition to *Chlamydia psittaci*, *Klebsiella pneumonia* was the most frequently identified bacteria in RICU patients, whereas *Streptococcus pneumonia* was most commonly identified in general ward patients. *Candida albicans* was the most widespread fungus, affecting 2 patients in RICU and 2 patients in the general ward. *Human betaherpesvirus 7* was the most prevalent virus, detected in 1 patient in the RICU and 3 patients in the general ward.

### 3.5 Treatments

**Figure 6:**
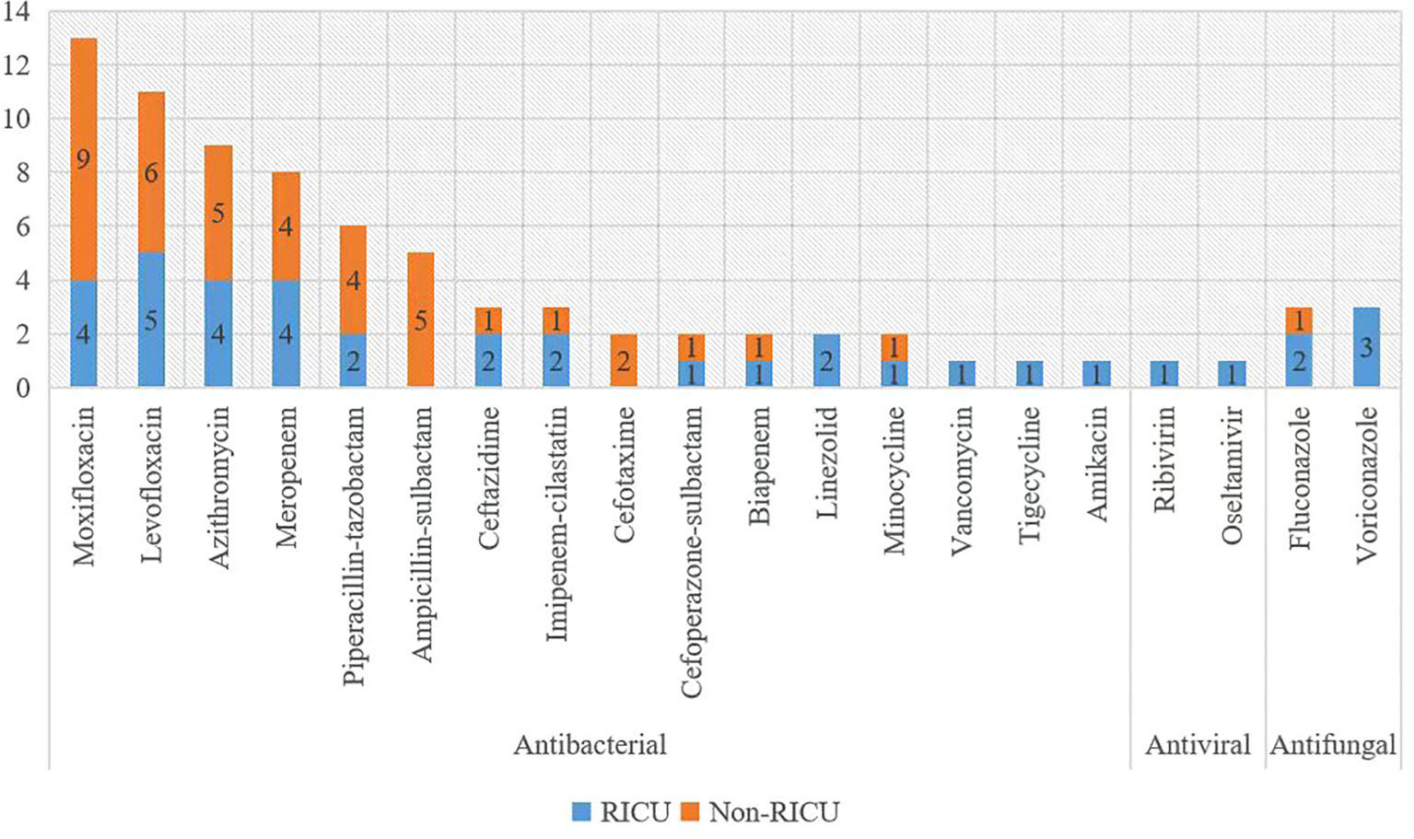
Antibiotics used for treatment. The x-axis displays Antibiotics, and the y-axis shows the case number.

All patients underwent treatment for psittacosis pneumonia. They received antibiotic therapy using either moxifloxacin or levofloxacin, following the community-acquired pneumonia management guidelines (Mandell et al., 2007; Balsamo et al., 2017). In case of viral infections, antiviral therapy was applied, in case of fungal infections, antifungal therapy was added, and in case of bacterial infections, antibiotics were applied accordingly (**Figure 6**).

## 4 Discussion

In this study, we investigated the prognostic value of CAR in assessing the severity of psittacosis pneumonia disease. Because changes in albumin and CRP levels are not observed simultaneously in the patients (Rathore et al., 2022), it has been suggested that the use of CAR could better correlate with the protein level and have great potential as a prognostic factor in patients with infected disease.

The mean age of the patients in this study was 62 years, male patients accounted for 70.4%, which was is similar to the results reported by Anbing Zhang (Zhang et al., 2022). Contact with birds or poultry is regarded as the main risk factor of psittacosis pneumonia and 12 patients (44.4%) in this study had a clear history of contact with poultry or birds. Therefore, patients with pneumonia should be asked about their poultry contact history because it was very important for the diagnosis of psittacosis pneumonia. A recent report also found that the transmission of psittacosis could occur by human-to-human transmission, even by asymptomatic carriers or by healthcare workers (Zhang et al., 2022).

It was found that the most prominent clinical symptom of these patients was fever, which occurred in all patients and most of them had high fever even over 39.1°C. Other common symptoms of psittacosis pneumonia include wheezing, cough, expectoration, chest tightness, fatigue and other flu-like symptoms. Patients treated on RICU were more likely to experience wheezing (88.9% vs 33.3%, P=0.013) and chest tightness (88.9% vs 33.3%, P=0.013) than general ward which implied that these two symptoms indicated more severe illness. In addition to the typical symptoms mentioned above, there were also some atypical symptoms including haziness of spirit-mind, myalgias and so on.

In this study, it was found that chest imaging lesions in psittacosis pneumonia patients were mostly consolidation and patchy shadowing with the air bronchogram sign and ground-glass opacities. Several imaging features, such as consolidation of the chest on chest CT bronchogram, have been reported (Gao et al., 2023). The other study reported that most patients have bilateral, mid-lower lobe, or multilobar lesions, usually presenting as consolidation, and have pleural effusions (Jiang et al., 2022; Ni et al., 2023). According to our study, 48.1% of patients had lesions involving the right lobe, 33.3% of patients had lesions involving the left lobe, and 18.5% of patients (4 patients in RICU and 1 patient in Non-RICU) had lesions involving bilateral lobes. The percentage of patients with pleural effusion was significantly higher in the RICU than in the general ward (88.9% vs.38.9%, P=0.019). It suggested that bilateral lung lesions and pleural effusion were mainly observed in severe cases.

It is worth noting that our laboratory analysis shows that the average white blood cell count was normal, which was consistent with the result reported by Vande Weygaerde Y that the white blood cell count was significantly lower in patients with psittacosis pneumonia compared with other pneumonia types (Vande Weygaerde et al., 2018). The C-reactive protein, PCT, IL-6, ALT AST and LDH were significantly elevated in laboratory tests, and albumin, PaCO_2_ were decreased than normal. PCT and CRP levels were more elevated in the RICU group than in the Non-RICU group. The other studies have found that significant increase in LDH level may indicate the severity of the disease, and may be predictor of severe pneumonia, and high LDH level is independent risk factor for severe psittacosis pneumonia (Su et al., 2021; Yang et al., 2022). However, no obvious difference in ALT, AST, LDH and IL-6 levels was found between RICU and Non-RICU group patients in our study. This finding needs to be further investigated by including more cases in the future.

Albumin was significantly decreased in RICU than Non-RICU, which may indicate the severity of psittacosis pneumonia. The mechanisms for low serum albumin levels are not well understood. However, the mean time from onset of psittacosis pneumonia to hospital admission is usually low (6.26 ± 4.20 days), which is less than the half-life of serum albumin (3 weeks), suggesting that hypoalbuminemia may be less likely to result from decreased albumin synthesis from the liver in severe psittacosis pneumonia. Further analysis found that albumin was not significantly correlated with hospitalization days (r=-0.3472, P=0.076). Similarly, the average BMI of the patients in our study is 25.25 ± 3.48 kg/m^2^, it can be assumed that poor nutrition (BMI<18.5kg/m^2^) may be less likely to cause the development of hypoalbuminemia. Thus, capillary permeability increased by inflammation can better explain for hypoalbuminemia in psittacosis pneumonia patients. This hypothesis was consistent with the results reported by Sawai Singh Rathore (Rathore et al., 2022). The PaCO_2_ level was significantly lower in RICU patients than in Non-RICU patients, possibly due to wheezing. Hypoxemia caused by inflammation leads to liver injury in most patients and kidney injury in some patients.

An elevated serum CAR reflects increased serum CRP and/or decreased serum albumin concentrations, high CRP and low albumin levels are associated with an increased risk of psittacosis pneumonia. A lot of studies have pointed out that CAR plays a certain role in the evaluation and prognosis of heart disease (Duman et al., 2019), cancer (Keinänen et al., 2021; Liao et al., 2021; Alkurt et al., 2022), stroke (Du et al., 2022), and some infectious diseases (Baykiz et al., 2022). A recent study concluded that CAR predicts in-hospital mortality risk in sepsis patients (Zhou et al., 2023). In our study, CAR was significantly higher in the RICU group than in the Non-RICU group (9.41 vs 4.05, P=0.017). This result was accompanied by higher intubation and ventilator support (33.3% vs 0.0%, P=0.029). Logistic regression analysis indicated that CRP (OR 1.01; 95% CI 1.00–1.03, P=0.016) and CAR (OR 1.49; 95% CI 1.07–2.06, P=0.017) were risk factors for prolonged hospitalization (> 14 days). Collectively, these findings indicate that CAR is a readily available and objective inflammatory biomarker for systemic inflammatory psittacosis pneumonia.

Through mNGS testing, we found that not all patients had *Chlamydia psittaci* infection only, some patients were also infected with other pathogens (**Figure 5**). Their treatment was not the same because we also had treatments that targeted other pathogens (**Figure 6**). We found that the number of pathogen sequences detected by mNGS in BALF was more compared with that in blood (there were two cases diagnosed through mNGS testing in both BALF and blood, the number of pathogen sequences were 8468 vs 69, 2207 vs 22, respectively), which is consistent with the result reported by Wei Gu et al (Gu et al., 2021). This suggests that if *Chlamydia psittaci* is detected in blood and contamination is ruled out, it may indicate the presence of an infection in the patient. However, this requires further validation and should not be ignored simply because of the low number of sequences. Further validation is required to confirm this, and the low number of sequences should not be used as a reason to ignore the result. In the case of patient P20, although only one read of *Chlamydia psittaci* was detected in the blood sample, the diagnosis of a *Chlamydia psittaci* infection was confirmed following a comprehensive examination and diagnostic process. In short, the interpretation of all mNGS results should not only be based on read cutoffs alone but also combined with the clinical context. It is necessary to ascertain whether the clinical manifestations, poultry contact history, other laboratory test results, imaging changes, and the efficacy of medication are consistent with the pathogens detected by mNGS.

To our knowledge, this is the first research to explore the relation between CAR and Chlamydia psittacosis pneumonia among adults of all genders. However, the research has several limitations. First, this is a retrospective study that included only 27 cases of psittacosis pneumonia. This small sample size is unable to explore all relevant features of psittacosis pneumonia. Second, most patients were diagnosed by mNGS, however, not all patients can afford mNGS. Clinically, the traditional culture method is not an effective means of detecting the related pathogens directly from the specimens due to the intracellular nature of *Chlamydia psittaci*. Despite the absence of *Chlamydia psittaci*-specific PCR and serological tests in our laboratory, the patient’s condition improved following treatment for psittacosis pneumonia based on the results of mNGS. In addition, detailed assessments of pneumonia severity, such as the pneumonia severity index and the CURB-65 score, were not available.

In conclusion, elevated serum CAR was associated with the severity of psittacosis pneumonia. It can be used as a simple and practical tool for clinicians to identify the severity of Chlamydia psittacosis pneumonia in a timely and accurate manner. Further research is needed to replicate this finding in a larger sample and to assess the potential value of CAR in the prognostic treatment strategies and follow-up strategies for psittacosis pneumonia.

## Data availability statement

The original contributions presented in the study are included in the article/**Supplementary Material**. Further inquiries can be directed to the corresponding authors.

## Ethics statement

The studies involving humans were approved by Human Research Committee at the Shanghai Fifth People’s Hospital, Fudan University. The studies were conducted in accordance with the local legislation and institutional requirements. The participants provided their written informed consent to participate in this study.

## Author contributions

TS: Conceptualization, Data curation, Funding acquisition, Investigation, Methodology, Writing – original draft, Writing – review & editing. YY: Data curation, Methodology, Writing – review & editing. YS: Data curation, Methodology, Writing – review & editing. MS: Data curation, Methodology, Writing – review & editing. YoD: Data curation, Methodology, Writing – review & editing. MeZ: Data curation, Methodology, Writing – review & editing. ZM: Data curation, Methodology, Writing – review & editing. YiD: Data curation, Methodology, Writing – review & editing. JF: Data curation, Methodology, Writing – review & editing. MoZ: Data curation, Methodology, Writing – review & editing. FG: Data curation, Methodology, Writing – review & editing. QZ: Data curation, Methodology, Writing – review & editing. RW: Conceptualization, Funding acquisition, Supervision, Validation, Writing – review & editing. ZJ: Conceptualization, Funding acquisition, Supervision, Validation, Writing – review & editing.
