# Supplementary material for: Serum C-reactive protein to albumin ratio as a potential risk indicator of pneumonia caused by Chlamydia psittaci: a multicenter retrospective study
Source: Front Cell Infect Microbiol. 2024 Jun 26;14:1371625. doi: 10.3389/fcimb.2024.1371625 (PMC11233447; doi:10.3389/fcimb.2024.1371625)
Supplement: Supplementary file 1 [file Table_1.docx]

Table S1 mNGS results from 27 patients.

| **Patient** | **Group** | **mNGS sample type** | ***Chlamydia psittaci* reads** | **Other Pathogens detected by mNGS (reads)** |
| --- | --- | --- | --- | --- |
| P1 | Non-RICU | BALF | 306 | *Human alphaherpesvirus 1* (3) |
| P2 | RICU | BALF/Blood | 8468 / 69 | *Human betaherpesvirus 7* (6) |
| P3 | RICU | BALF/Blood | 2207 / 22 | *Candida albicans* (143), *Aspergillus flavus* (1) |
| P4 | RICU | Sputum | 10 | *Acinetobacter baumannii* (9984) |
| P5 | RICU | BALF | 45 | / |
| P6 | Non-RICU | BALF | 20 | *Streptococcus Pneumoniae* (346) |
| P7 | RICU | BALF | 33 | *Nakaseomyces glabratus* (796), *Candida albicans* (244), *Human betaherpesvirus 5* (1) |
| P8 | Non-RICU | BALF | 112 | *Mycobacteroides abscessus* (2), *Human betaherpesvirus 6B* (303) |
| P9 | Non-RICU | BALF | 411 | *Streptococcus Pneumoniae* (2474), *Streptococus mitis* (5705) |
| P10 | Non-RICU | BALF | 53 | *Human alphaherpesvirus 1* (1), *Human gammaherpesvirus 4* (9), *Human betaherpesvirus 7* (6) |
| P11 | Non-RICU | BALF | 44 | *Lautropia mirabilis* (2287), *Pneumocystis jirovecii* (1), *Human gammaherpesvirus 4* (1), *Human betaherpesvirus 7* (2) |
| P12 | Non-RICU | BALF | 6 | *Mycobacteroides abscessus* (10) |
| P13 | Non-RICU | BALF | 528 | *Mycobacteroides abscessus* (20), *Enterococcus avium* (838) |
| P14 | Non-RICU | BALF | 18 | *Aureobasidium melanogenum* (10) |
| P15 | RICU | BALF | 211 | *Klebsiella pneumoniae* (10243), *Pseudomonas aeruginosa* (752) |
| P16 | RICU | BALF | 11327 | *Klebsiella pneumoniae* (132) |
| P17 | Non-RICU | BALF | 21 | *Streptococcus Pneumoniae* (5) |
| P18 | Non-RICU | BALF | 7 | *Cryptococcus neoformans* (6) |
| P19 | Non-RICU | BALF | 101 | *Human betaherpesvirus 7* (45) |
| P20 | RICU | Blood | 1 | *Human gammaherpesvirus 4* (1) |
| P21 | Non-RICU | BALF | 5 | *Streptococcus Pneumoniae* (135593), *Candida albicans* (85041) |
| P22 | Non-RICU | BALF | 565 | *Streptococcus Pneumoniae* (170243), *Haemophilus parainfluenzae* (818) |
| P23 | Non-RICU | Blood | 20 | / |
| P24 | Non-RICU | BALF | 51 | *Staphylococcus argenteus* (224), *Enterococcus Faecium* (13), *Alcaligenes xylosoxidans* (5) |
| P26 | Non-RICU | BALF | 2677 | *Finegoldia magna* (42), *Haemophilus parainfluenzae* (13), *Candida albicans* (885), *Aspergillus fumigatus* (3), *Mycobacteroides abscessus* (29) |
| P27 | RICU | BALF | 530 | *Klebsiella pneumoniae* (9) |
| P28 | Non-RICU | Sputum | 13 | *Streptococcus Pneumoniae* (552), *Acinetobacter baumannii* (45) |
